# Anatomical study of single incision contralateral C7 nerve transfer through subdural pathway

**DOI:** 10.3389/fnana.2024.1470913

**Published:** 2024-10-30

**Authors:** Long Yao, Zhengcun Yan, Xiaodong Wang, Jiaxiang Gu, Hongjun Liu, Hengzhu Zhang

**Affiliations:** ^1^Department of Neurosurgery, Clinical Medical College of Yangzhou University, Yangzhou, China; ^2^Hand and Foot Microsurgery, Clinical Medical College of Yangzhou University, Yangzhou, China

**Keywords:** spinal canal, subdural pathway, C7 nerve, nerve transfer, anatomic

## Abstract

**Objective:**

To explore the feasibility of single incision C7 nerve transfer surgery through the subarachnoid pathway on the healthy side through anatomical research.

**Method:**

Four fresh frozen cadaver specimens were used for the study. Observe and measure the length of C7 nerve root fibers. Divide the front root into 3 bundles and the rear root into 5 bundles.

**Result:**

The C7 nerve has a filamentous structure, arranged symmetrically on both sides, and the length of the root fibers gradually shortens from top to bottom. The length of the left anterior root decreased from (12.25 ± 0.68) mm to (9.75 ± 1.40) mm, the length of the right anterior root decreased from (12.95 ± 1.49) mm to (10.00 ± 2.00) mm, the length of the left posterior root decreased from (15.63 ± 1.55) mm to (12.38 ± 0.71) mm, and the length of the right posterior root decreased from (15.48 ± 1.37) mm to (12.30 ± 0.90) mm. The distance from the exit of the C7 nerve from the dura mater to the fusion site in 4 specimens was (10.98 ± 1.21) mm on the left and (10.98 ± 1.391) mm on the right. All four specimens have completed nerve bundle anastomosis.

**Conclusion:**

From an anatomical perspective, it is feasible to anastomose the healthy side C7 nerve with the affected side root fibers in the dorsal bundle of the spinal cord after cutting off the dura mater.

## Introduction

In China, stroke is characterized by high incidence rate, high disability rate, high recurrence rate and high mortality. Among them, hemiplegia is the main factor affecting the prognosis of stroke patients. Finding a method that can improve both hemiplegic limb movement and relieve spasms has become one of the important topics in the medical field. This article proposes a surgical approach for the transfer of the contralateral C7 nerve through a single incision intraspinal subdural pathway through microanatomy and simulated surgery of the C7 nerve in the spinal canal. To provide theoretical and clinical basis for the feasibility of contralateral C7 nerve transfer surgery in the spinal canal.

## Methods

### Ethical procedures

This study was conducted in accordance with the principles of the Helsinki Declaration. The cadaver specimens used in this study were provided by the Anatomy Laboratory of Yangzhou University School of Clinical Medicine and were reviewed and approved by the Ethics Committee of Yangzhou University School of Clinical Medicine. Approval number: J-20200105. Prior to the publication of this study, the Anatomy Laboratory of Yangzhou University School of Medicine had obtained written informed consent from all participants. This study protected the anonymity of all patient information, and no one except the researchers could identify the patient’s identity.

### Selection of specimens

Four fresh frozen adult corpses. Two males and two females, aged (40.3 ± 14.1) years old (24–58 years old). Four cadaver specimens had no previous history of cervical spine surgery, and the cervical spine and its attached ligaments, muscles, and other tissues were preserved intact, with intact skin.

### Equipment and instruments

Grinding Drill, Lamina Rongeur, Nerve Stripping Ion, Vascular Forceps, Microscope, Neuroendoscope, 12–0 Suture, Titanium Alloy Nail Plate, Electronic Digital Vernier Caliper, etc.

### Operation process

**Figure 1:**
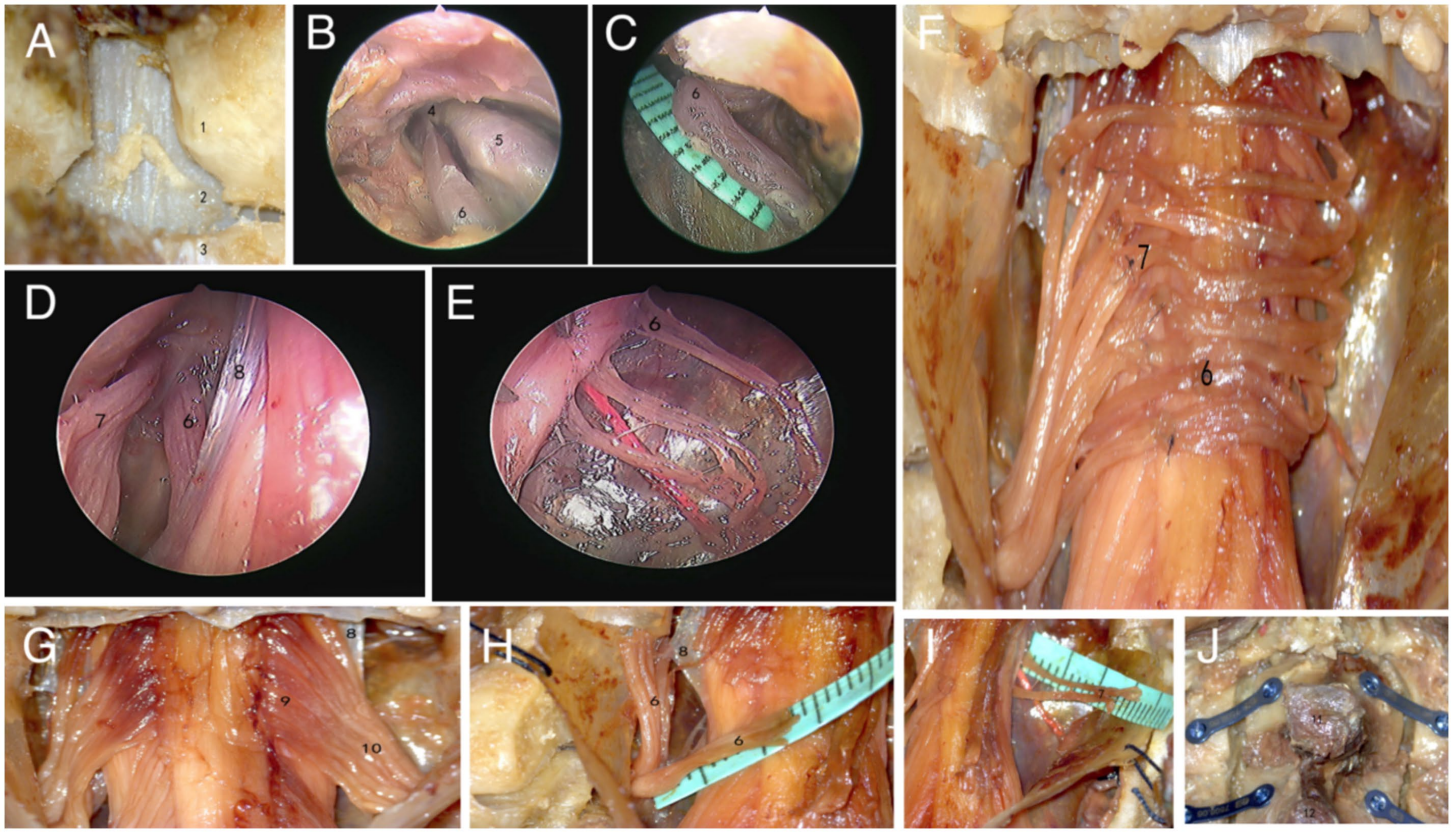
Microscopic and endoscopic observation results. (A–E) Firstly, grind open the vertebral lamina, observe and measure the length from the anterior root of the epidural C7 nerve to the fusion site using a neuroendoscope. Then, cut open the dura mater and observe the distribution of the anterior and posterior roots of the C7 nerve (1.C6 inferior articular process, 2.dura mater, 3.C7 superior articular process, 4.C7 spinal nerve, 5.Spinal ganglia, 6. anterior root of C7 nerve, 7.posterior root of C7 nerve, 8.dentate ligament). (F–I) Observe and measure the lengths of the anterior and posterior roots of bilateral C7 nerves under a microscope, and complete nerve anastomosis (9.C7 nerve fasciculus, 10.C7 posterior root nerve sub bundles). (J) Fix the C6 and C7 vertebral plates with titanium plates and screws (11.C6 spinous process, 12.C7 spinous process).

After completely thawing the specimen, take a prone position and use the C7 spinous process as a marker point to longitudinally cut through the skin and muscles, fully exposing the C6 and C7 vertebral lamina. Use a grinding drill to grind away some of the C6 spinous processes from the center, removing 1/3 of the C6 lower articular process and 1/3 of the C7 upper articular process, fully exposing the dura mater (Figure 1A). Observe the route of C7 nerve within the spinal canal under neuroendoscopy, and measure the length of C7 nerve from the dura mater to the fusion site of the anterior and posterior root nerves of the intervertebral foramen (Figures 1B,C). Cut open the dura mater and observe the anatomical position relationship of the anterior and posterior roots on both sides as they exit the dura mater using a neuroendoscope, as well as the branching status of the anterior and posterior root filaments (Figure 1D). Grind open the bilateral vertebral lamina of C6 and C7 with a grinding drill, cut open the arachnoid membrane, and separate the anterior and posterior root filaments of the C7 nerve under a microscope. Measure the distance from the origin point of the anterior and posterior roots to the dura mater (Figures 1H,I). Divide the anterior root into 3 bundles and the posterior root into 5 bundles. The posterior root completes the level anastomosis of nerve sub bundles to nerve sub bundles, and the anterior root completes the level anastomosis of nerve sub bundles to nerve small bundles (Figures 1E,F). Finally, suture the arachnoid and dura mater, fix the C6 and C7 vertebral plates with titanium plates (Figure 1J), and suture the muscles and skin layer by layer.

## Statistics

The measured data are expressed as mean ± standard deviation (x ± s), which is expressed by SPSS 17.0 statistical software for analysis.

## Results

### Anatomical measurement results

The length measurement of C7 on the left and right sides of four specimens from the dura mater to the fusion site is shown in Table 1. The measurement of C7 nerve root filament length is shown in Table 2.

**Table 1:** Distance from the left and right C7 nerves out of the dura mater to the fusion site.

Position	Length(mm)
Left	10.98 ± 1.209
Right	10.98 ± 1.391
T value	0.000
*p* value	>0.9999

**Table 2:** Measurement of C7 nerve root filament.

Position	1st Bundle	2nd Bundle	3rd Bundle	4th Bundle	5th Bundle
Left anterior root	12.25 ± 0.676 mm	11.10 ± 0.859 mm	9.75 ± 1.401 mm		
Right anterior root	12.95 ± 1.493 mm	11.90 ± 1.386 mm	10.00 ± 2.002 mm		
Left posterior root	15.63 ± 1.554 mm	15.00 ± 1.463 mm	13.93 ± 1.118 mm	13.33 ± 1.075 mm	12.38 ± 0.709 mm
Right posterior root	15.48 ± 1.372 mm	14.83 ± 1.360 mm	14.05 ± 1.088 mm	13.18 ± 1.153 mm	12.30 ± 0.898 mm

### Observations results

Opening the vertebral lamina, it can be seen that the C7 nerve runs outwards and downwards after exiting the dura mater in the spinal canal. Opening the dura mater, it can be clearly observed that the C7 nerve has a filamentous structure, and the toothed ligament separates the anterior and posterior roots. The nerve root filaments attach to the connective tissue beneath the soft membrane. From the attachment point of the root filament to the spinal cord, it is sequentially divided into nerve fasciculus and nerve fasciculus outwards and downwards. There is no clear boundary point between the nerve fasciculus and the nerve sub fasciculus, and its approximate boundary point is located at the outer edge of the dentate ligament (Figure 1G). Separate the nerve fasciculus, and pull it to show greater toughness, accompanied by a root artery, with each nerve fasciculus relatively independent of each other. The number of nerve fascicles in the anterior root is 6–9, and the number of nerve fascicles in the posterior root is 8–12. The nerve bundles gradually converge into nerve sub bundles, which are closely connected but easy to separate. The number of nerve sub bundles in the anterior root is 3–5 bundles, and the number of nerve sub bundles in the posterior root is 5–8 bundles. After the nerve sub bundles in the anterior and posterior roots converge again, they, respectively, penetrate the dura mater.

### Simulated surgery

All four specimens underwent simulated surgical procedures under the assistance of neuroendoscopy and microscopy. Among them, two specimens completed one-on-one anastomosis of the anterior roots, while two specimens showed excessive anastomosis tension in the anterior roots. We chose to anastomose the first bundle of the healthy anterior roots with the third bundle of the affected anterior roots, and the third bundle of the healthy anterior roots with the first bundle of the affected anterior roots. None of the four specimens showed any further separation of the dura mater to extend the anterior root nerve bundle, while the posterior roots underwent one-on-one anastomosis at the level of nerve sub bundles to nerve sub bundles. When suturing the arachnoid membrane, there is a tearing phenomenon of the arachnoid membrane, and the dura mater can be completely sutured.

## Discussion

Neurotransposition is currently the main method for treating pre ganglionic and root avulsion injuries of the brachial plexus (Tsuyama et al., 1969; Kotani et al., 1971; Brunelli and Monini, 1984; Oberlin et al., 1994; Gu et al., 1987; Gu, 2004). The nerves available for transposition include intercostal nerves, accessory nerves, cervical plexus nerves, phrenic nerves, and the contralateral C7 nerve. The brachial plexus nerve belongs to the peripheral nerve, and damage to the peripheral nerve can be replaced by nerve displacement. However, after a stroke, it will cause permanent damage to the function of the damaged area of the brain, which cannot be replaced. Liu et al. (2020) studied the NeuroD1 gene to convert glial cells into neuronal cells, which can essentially repair damaged brain cells. However, this research is still in the animal experimental stage. How to restore movement of hemiplegic limbs has become one of the important issues in today’s medical field. In addition to conservative treatments such as traditional Chinese medicine, medication, and rehabilitation training, surgical treatment has gradually become the main clinical treatment method due to its precise clinical efficacy and fast recovery. At present, the main surgical methods for treating spastic hemiplegia include stereotactic surgery, carotid endarterectomy, selective peripheral nerve transection of limbs, selective posterior root nerve transection of spinal nerves, and the contralateral C7 nerve transfer surgery.

In 1986, Gu (2004) successfully applied the contralateral C7 nerve transfer method for the first time to treat brachial plexus root avulsion injury. At present, this surgery is not only widely used in the repair of brachial plexus nerve injuries, but also provides a new treatment plan for patients with spastic hemiplegia of the upper limbs (Seruya, 2018; Gu et al., 2002), achieving control of both limbs by one brain. However, this surgery is limited to improving the motor function of the upper limbs and has little effect on hemiplegia of the lower limbs. However, there are also literature reports that while solving upper limb spasticity, the lower limbs can also be correspondingly improved (AlHakeem et al., 2020). Hua et al. (2015) conducted a 2-year follow-up on 12 patients with central nervous system injury (Group A underwent the contralateral C7 nerve transfer surgery, while Group B only received rehabilitation treatment). The results showed that all 6 patients in Group A experienced relief of affected upper limb flexor muscle spasms and improvement in motor function, while no significant motor recovery or deformity relief was observed in the 6 patients in Group B. Zheng et al. (2018) followed up 36 patients with unilateral arm paralysis for more than 5 years (18 patients received C7 nerve transplantation and rehabilitation treatment, and 18 patients received rehabilitation treatment alone) for 12 months and found that C7 nerve transfer from the healthy side to the paralyzed side significantly improved upper limb function and reduced spasticity compared to simple rehabilitation treatment, and developed physiological connections between the ipsilateral cerebral hemisphere and the paralyzed hand.

### The rationality of intraspinal nerve anastomosis

The C7 nerve is a mixed nerve of afferent and efferent nerves. Cutting off the C7 nerve on the affected side not only cuts off the efferent of *γ* motor neurons, but also cuts off the efferent of Ia/II sensory neurons, and at the same time cuts off the efferent of *α* motor neurons, weakening the contraction of the intraspinatus and extraspinatus muscles and relieving spasms. There is sufficient fiber volume in both the anterior and posterior thighs of the C7 nerve (Lu et al., 2004), and the transection of the C7 nerve on the healthy side has no or only temporary effects on the healthy side (Jiang et al., 2011). After being relocated to the affected side, the affected side re accepts the brain’s role in the posture regulation system, which to some extent inhibits spasms. This resolves spasms not only from the γ loop (small loop) (Sehgal and McGuire, 1998), but also from the cortex-spinal cord-cortex loop (large loop) (Wei et al., 2008; Yu and Xu, 2004). Guo et al. (2021) found that C7 nerve anastomosis requires whole root anastomosis, and anastomosing only the anterior root or posterior root can affect cortical remodeling, thereby affecting surgical outcomes. The spinal nerves are divided into anterior and posterior roots within the spinal canal, which converge and re fuse into the C7 nerve trunk within the intervertebral foramen. The anterior roots anastomose with the anterior roots, and the posterior roots anastomose with the posterior roots, ensuring the forward conduction of the nerves and further improving the accuracy of nerve conduction. However, the efficiency of this surgical approach has not been confirmed yet. Our research team will demonstrate in subsequent animal experiments that the conduction efficiency of anterior root anterior root and posterior root posterior root anastomosis is superior to spinal nerve spinal nerve anastomosis.

### Surgical treatment of central hemiplegia with contralateral C7 nerve transfer

At present, the surgical methods that have been clinically applied are the anterior esophageal posterior approach through the vertebral body (Zhao et al., 2021; Yan et al., 2022a) and the modified posterior approach through the vertebral body (Wu et al., 2020; Yan et al., 2022b), both of which involve nerve anastomosis outside the spinal canal. Compared to this, our surgical approach has greater advantages: ① Adopting a single incision in the neck and back, avoiding changes in the patient’s position during the surgery. The incision is smaller than the anterior approach and has less trauma to the body. ② Operating within the spinal canal avoids searching for C7 nerves and opening the pathway from the healthy side to the affected side, shortens the surgical time, and the anterior and posterior roots are anastomosed separately to further ensure positive nerve conduction. At the same time, the technical requirements for the surgeon are not high, making it easy to popularize and promote.

### Problems and solutions in spinal canal nerve anastomosis surgery

① Length of nerve bundle: the healthy anterior root needs to detour from behind to reach the affected side. The observation results of this study demonstrate that the left anterior root length can be reduced from (12.25 ± 0.68) mm to (9.75 ± 1.40) mm, and the right anterior root length can be reduced from (12.95 ± 1.49) mm to (10.00 ± 2.00) mm, both of which can complete nerve anastomosis. However, due to anatomical differences, we need to measure the sagittal diameter of the C6/7 spinal cord and the length of the C7 nerve anterior root before surgery to ensure that the length of the healthy anterior root separated is at least half of the spinal cord circumference. After calculation, we will evaluate whether the patient is suitable for this surgery. The first bundle has sufficient detour length, while the third bundle requires a longer length. If the length is sufficient, the root filaments are matched one-on-one, which is in line with anatomical anastomosis. If the tension after root silk anastomosis is high, cross anastomosis can be chosen, or sufficient length of the anterior root can be further separated to the fusion of the anterior and posterior roots before anastomosis. ② Cerebrospinal fluid leakage: intraspinal nerve anastomosis surgery requires opening the dura mater and arachnoid membrane to separate the nerves, so there is a high risk of cerebrospinal fluid leakage. ③ Separation of nerve bundles: All nerve bundles have accompanying blood vessels, especially the anterior root artery, which should be separated with extra caution. During the process of separating nerve bundles, excessive clamping should be avoided. Due to the small diameter of nerve bundles, surgical operations need to be performed under neurophysiological monitoring to prevent and reduce iatrogenic nerve and spinal cord injuries (Julio et al., 2015). ④ How many nerves are divided: in this study, the posterior roots of bilateral C7 nerves were evenly divided into 5 bundles. Firstly, dividing into 5 bundles has the least destructive effect on the nerve bundle membrane; secondly, after dividing into 5 bundles, each bundle has a diameter of approximately 1.0 mm, and is easy to anastomose with a 12–0 suture needle. If there are too many separated nerve bundles, on the one hand, it will increase the difficulty of nerve anastomosis and prolong the surgical time; On the other hand, due to the partial association between nerve sub bundles, the separation is too fine to easily cut off these connections. If the separation is too small, too coarse, and not precise enough, the goal of fine conduction cannot be achieved. After comprehensive analysis, each side was evenly divided into 5 bundles in this study; similarly, divide the front root into three bundles. This is basically consistent with the research results of Bonnel (1984) and Peng et al. (2006). With the rapid development of ultra microsurgery, nerve anastomosis will become increasingly refined. ⑤ Suture method of nerve: After comprehensive analysis, this study used the epineurial suture method. ⑥ Effect of neck activity on nerve tension: the cervical spine carries the weight of the entire head and has a high degree of activity, all of which stretch the nerves. The research results of Ishii et al. (2006) and Mao et al. (2016) suggest that although head and neck activity can drive vertebral movement, the rotation angles of C6 and C7 are relatively small, resulting in minimal traction on the nerves in the intervertebral foramen. ⑦ Changes in spinal structural integrity and stability: the bone structure and muscle ligament complex at the back of the spine significantly affect the stability of the spine (Liu et al., 2007). In addition, due to the opening of the bilateral vertebral lamina of C6 and C7, the stability of the posterior column of the spine is also reduced. This study used titanium connecting plates and titanium screws for internal fixation of the spinous process lamina complex after surgery, with the bilateral lateral masses remaining intact, making the fixation more secure and facilitating postoperative movement adjustment.

In summary, the results of this study confirm that it is feasible for the controlateral C7 nerve to be anastomosed with the affected root filament in the dorsal bundle of the spinal cord after being cut off under the dura mater, and there is no need for bridging the nerve. However, there are still certain shortcomings in this study. ① The number of specimens studied in this experiment is relatively small, and they are frozen cadaver specimens, which have certain differences from the physiological state of living organisms. ② This study is only an anatomical study and has not yet conducted *in vivo* animal experiments. The functional recovery after nerve anastomosis still needs further experimental verification.

## Conclusion

From an anatomical perspective, it is feasible to anastomose the controlateral C7 nerve with the affected root filament in the dorsal bundle of the spinal cord after being cut off under the dura mater, providing theoretical and clinical basis for the feasibility of the contralateral C7 nerve intraspinal transfer surgery.

## Data Availability

The datasets presented in this study can be found in online repositories. The names of the repository/repositories and accession number(s) can be found in the article/Supplementary material.
