# Supplementary material for: Anatomical study of single incision contralateral C7 nerve transfer through subdural pathway
Source: Front Neuroanat. 2024 Oct 30;18:1470913. doi: 10.3389/fnana.2024.1470913 (PMC11557413; doi:10.3389/fnana.2024.1470913)
Supplement: Supplementary file 1 [file Table_1.DOCX]

**Extended Date Tab.1|Measuring data of left** anterior **root wire length (mm).**

|  | 1st bundle | 2nd bundle | 3rd bundle |
| --- | --- | --- | --- |
| Specimen 1 | 11.5 | 10.5 | 8.5 |
| Specimen 2 | 13.0 | 12.1 | 11.2 |
| Specimen 3 | 12.6 | 11.5 | 10.7 |
| Specimen 4 | 11.9 | 10.3 | 8.6 |

**Extended Date Tab.2|Measuring data of right** anterior **root wire length (mm).**

|  | 1st bundle | 2nd bundle | 3rd bundle |
| --- | --- | --- | --- |
| Specimen 1 | 12.1 | 11.5 | 9.1 |
| Specimen 2 | 15.1 | 13.9 | 13.0 |
| Specimen 3 | 11.8 | 10.7 | 9.0 |
| Specimen 4 | 12.8 | 11.5 | 8.9 |

**Extended Date Tab.3|Measuring data of left** posterior **root wire length (mm).**

|  | 1st bundle | 2nd bundle | 3rd bundle | 4th bundle | 5th bundle |
| --- | --- | --- | --- | --- | --- |
| Specimen 1 | 13.4 | 13.0 | 12.5 | 12.0 | 11.5 |
| Specimen 2 | 17.0 | 16.5 | 15.2 | 14.5 | 13.0 |
| Specimen 3 | 15.9 | 15.1 | 13.8 | 13.0 | 12.1 |
| Specimen 4 | 16.2 | 15.4 | 14.2 | 13.8 | 12.9 |

**Extended Date Tab.4|Measuring data of right** posterior **root wire length (mm).**

|  | 1st bundle | 2nd bundle | 3rd bundle | 4th bundle | 5th bundle |
| --- | --- | --- | --- | --- | --- |
| Specimen 1 | 15.1 | 14.5 | 14.1 | 13.4 | 12.8 |
| Specimen 2 | 17.5 | 16.8 | 15.5 | 14.7 | 13.3 |
| Specimen 3 | 14.8 | 14.3 | 13.7 | 12.5 | 11.7 |
| Specimen 4 | 14.5 | 13.7 | 12.9 | 12.1 | 11.4 |

**Extended Date Tab.5|C7 Length of nerve from dura mater to fusion (mm).**

|  | Left | Right |
| --- | --- | --- |
| Specimen 1 | 9.5 | 9.0 |
| Specimen 2 | 12.4 | 12.2 |
| Specimen 3 | 11.3 | 11.6 |
| Specimen 4 | 10.7 | 11.1 |
